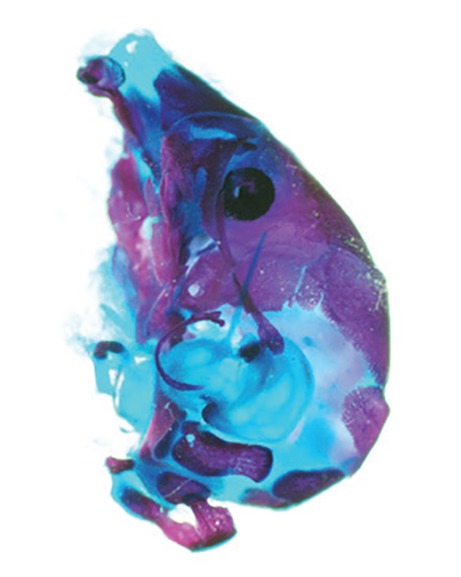# Insights into orofacial birth defects

**Published:** 2015-01

Cleft lip with or without cleft palate (CL/P) is one of the most frequent human congenital defects. Treatments for this condition are possible but can only partially rescue related speech and structural alterations, and require lifelong medical management. To find new treatments it is important to further understand the mechanisms of clefting development. A current view is that alterations in facial shape or fusion of facial prominences – the tissues that give rise to orofacial structures – might play a role. However, available mouse models can only partially recapitulate clefting phenotypes. Here, Trevor Williams and colleagues develop a new mouse model that bears mutations in the gene encoding transcription factor AP-2α (*Tfap2a*) – associated with human CL/P – and that shows full CL/P penetrance. By employing geometric morphometrics, the authors analysed facial growth and shape changes in control and mutant mice and found that, in mutants, clefting develops owing to altered facial morphology. Interestingly, mutants also exhibited a slight increase in fibroblast growth factor 8 (*Fgf8*) expression; decreasing *Fgf8* dosage in these mice could partially rescue CL/P. The study suggests that orofacial development is particularly sensitive to small changes in gene expression and that FGF signalling could be targeted as an early therapeutic intervention in CL/P. **Page 31**

**Figure:**